# Using Complex Networks to Characterize International Business Cycles

**DOI:** 10.1371/journal.pone.0058109

**Published:** 2013-03-04

**Authors:** Petre Caraiani

**Affiliations:** Institute for Economic Forecasting, Romanian Academy, Bucharest, Romania; Tel Aviv University, Israel

## Abstract

**Background:**

There is a rapidly expanding literature on the application of complex networks in economics that focused mostly on stock markets. In this paper, we discuss an application of complex networks to study international business cycles.

**Methodology/Principal Findings:**

We construct complex networks based on GDP data from two data sets on G7 and OECD economies. Besides the well-known correlation-based networks, we also use a specific tool for presenting causality in economics, the Granger causality. We consider different filtering methods to derive the stationary component of the GDP series for each of the countries in the samples. The networks were found to be sensitive to the detrending method. While the correlation networks provide information on comovement between the national economies, the Granger causality networks can better predict fluctuations in countries’ GDP. By using them, we can obtain directed networks allows us to determine the relative influence of different countries on the global economy network. The US appears as the key player for both the G7 and OECD samples.

**Conclusion:**

The use of complex networks is valuable for understanding the business cycle comovements at an international level.

## Introduction

There is a growing interest in the application of complex networks in economics, with the number of publications on this topic increasingly rapidly. Not surprisingly, most studies on this topic focused on stock markets. In this paper we first review the main results in the literature on complex networks which will serve as a background for our empirical analysis.

The paper by [1] was among the first to use correlation to construct networks from financial returns. Based on the constructed correlation matrix between the most traded stocks, he filtered the information using the minimum spanning tree, obtaining thus a final complex network A refinement of this work was done by [2], who based on the correlation matrix of stocks, filtered the information using the planar maximally filtered graph, obtaining a graph that contains more information than the minimum spanning tree.

The use of standard correlation to construct correlation matrices has been improved by [3] through the introduction of the partial correlation. By using a threshold to eliminate the lack of correlation as well as a ranking of the partial correlations, they sought to reveal the causality between the different stocks. This approach was shown to better reveal the dominating stocks in the New York Stock Exchange market. The stocks from the financial sector and from the investment services sub-sector are the most influential ones.

Following the challenges of the last financial crisis, [4] used data for S&P 500 between 1999 and 2010 to construct correlation networks, addressing thus the need to construct more relevant measures of stock market dynamics. They proposed a new system-level parameter, a so called index cohesive force. Based on this index, they could analyze the state of the market and the probability of a market crash.

A better understanding of the role of nodes in financial networks is due to [5] who proposed a new methodology to understand the relationship between nodes in financial networks which relies on the dependency network. Analyzing this new matrix in time for the case of Dow Jones Industrial Average components, they found that it can be used to identify particular crises episodes.

While the initial research on financial networks focused on individual case studies, recent research has also studied the relationship between different stock markets. For example, [6] used inter and intra correlations, the market index cohesive force as well as meta-correlations to analyze the dynamics of and relationships between main world capital markets. They found different patterns for Western and emerging Asian markets.

Obviously there are several good reasons for the focus on stock markets, including the high number of observations as well as their behavior (especially from the developed economies) close to the efficiency hypothesis (i.e. all the information is reflected in the price of a stock). The increasingly complex networks led to certain properties, like the known stylized fact that stocks tend to group together according to the sectors which they are part of.

A much less studied topic is that of the national economies or the global macroeconomic relationships using complex networks. Most of the existing research on this topic focuses on constructing the complex networks based on trade data, see [7] and [8] for two of the most representative studies. Global economic linkages were studied by [7] who used data on international trade. Based on the complex network statistics the work tried to explain the recent financial crises. They found that a network based approach can explain the contagious character of the financial crises in Mexico, Russia and South-East Asia.

The possibility of studying international crises in a network setting was proposed by [8]. The authors constructed a model of crisis-spreading which was applied on a complex network based on combined data on GDP and trade between the countries. Their main finding was that a country plays a more significant role in a financial crisis the larger the GDP and the bigger its connectivity at both global and local level.

In this study we analyze global business cycles using the tools and results from the network theory. According to the modern view, see [9], business cycles are understood as deviations from a growing trend. This is why, in practice, the business cycle component of a macroeconomic time series is derived based on extracting the trend, see the methodology section below. While much of the existing literature has been devoted to understanding national business cycles, either in the US or other developed and developing economies, there is a growing interest in understanding the international business cycles, starting with the work by [10]. Some effort has been dedicated to mapping of the so called stylized facts of international business cycles, i.e. the comovement between the different variables of national economies, see [11] or [12]. [12] analyzed the cross-correlations between the main macroeconomic variables of 20 industrialized countries; the most common finding are weak positive correlations.

A pertinent question is why such an approach would help us better understanding international business cycles. First, given the increasing globalization and integration of world economies, we would expect that there are increased linkages between the different national economies. A second effect is that a crisis within economy (especially a large one like US) can have wide effects on the global economy. The last housing market crisis is the classic example. Recent sovereign debt crisis has shown that even small open economies can have significant effects on the state of more developed economies due to the financial links between them. Thus a network based approach can help us better understand the role of countries taken as nodes as well as the linkages between them.

This paper approaches the modeling of macroeconomic dynamics at an international level using complex networks. Several contributions to existing literature are intended. First, we focus on GDP dynamics, studying the macroeconomic features with complex networks. Moreover, complex networks are constructed based on the correlations between the GDP of national economies. The concept of Granger causality (see below) is also used to construct complex networks, an approach used only very recently by [13]. With this approach we address the main shortcoming of both the Pearson correlation coefficient and partial correlations, (see [3] for a more extensive discussion.).

The paper is organized as follows. The methodology used throughout the analysis is described in the second section. The third section presents and discusses the empirical results. The last section draws conclusions and outlines some possible extensions of our work.

## Methods

Here, we begin with a presentation of the approaches to filter the series since they are not stationary in their initial form. The methods used to derive correlation networks are outlined. Then, we apply the minimum spanning tree to the correlation networks.

### Filtering the Macroeconomic Time Series

A sensitive issue in economics is to derive the cycle component of a series. This is necessary in the context of this paper as the GDP series contain a trend. A first and standard approach to filter a time series in order to eliminate the trend is to calculate the first difference. This approach is usually employed for financial time series too.

Given a time series *y_t_* for country *i*, the growth rates can be computed using the log differences as given below:
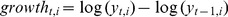
(1)There are, however, other standard approaches in economics, some of them accompanied by serious criticisms. We present and use later the Hodrick Prescott filter [14].

For each macroeconomic time series *y_t_* we perform a decomposition into its cycle component *c_t_* and trend component *µ_t_* based on an minimization process given below:
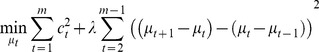
(2)Where “*m”* is the number of observation and *λ* is a smoothing parameter that controls the variability in the trend component *µ_t_*. The role of this filter is to identify the business cycle component by eliminating the trend, as the widely accepted definition of business cycles says that cycles are deviations from the growing trend.

There are many other filtering methods employed in economics, like band pass filters, see [15] for a relevant example, or the more modern wavelet approach, see [16] for an early use in economics; however we stick in this study only to the standard approaches. A key reference on the effects of using different filters on the business cycles properties is due to [17].

### Correlation Based Networks

There are different ways to construct networks from data series. One of the most popular is the Pearson correlation coefficient. A correlation matrix R is constructed using the standard correlation coefficient, as follows:
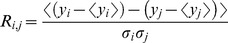
(3)With 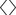 standing for the average and σ the standard deviations of the series *y_i_* and *y_j_*.

The resulting R matrix of dimensions NxN (where N is the number of countries involved in the analysis) corresponds to a weighted undirected network, with the weight of a link given by the correlation between the GDP of the different countries, namely by *ρ_ij_*. This network usually needs further filtering in order to eliminate the spurious links. In our paper we set to 0 the correlations which are smaller than 0.3 (a standard threshold in the literature).

A major issue with correlation is that it cannot detect causality, although, of course, it signals comovement. In the literature on complex networks, some attempts have been made to correct this. Various measures have been proposed, e.g., imposing thresholds for correlation, using partial or weighted correlation which try to eliminate spurious correlations. However, up to this moment, except for [13], we are not aware of constructing complex networks which really seek causality in a statistical sense.

### Granger Causality-based Networks

In the construction of the network we propose the use of Granger causality as a route to studying causality. Initially developed in the context of economics, it has been recently used in other fields such as neuroscience.

The so called causality test, as developed by Granger (1969) [18], is basically a test of whether a certain variable helps forecasting the future values of another variable.

The following equations are used to apply the test:
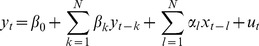
(4)

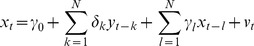
With *y_t_* and *x_t_* the two variables in a causal relationship, *u_t_* and *v_t_* uncorrelated disturbances, *k* and *l* the number of lags for each equation. The null hypothesis *H_0_* and the alternative hypothesis are given by *H_1_*.

*H0*: *α_l_ = 0* for any *l* and *δ_k_ = 0* for any *k.*

*H1*: *α_l_ ≠ 0* and *δ_k_ ≠ 0* for at least some *l* and/or *k*.

If α_l_ is statistically significant, then we say that *x* Granger causes *y*. If any *δ_k_* is different from zero, we say that *y* causes *x* in a Granger sense. We can also have bilateral causality.

Based on a resulting Granger causality matrix of relationships between countries, we derive a network following certain rules. These rules, in a realistic way, although not without some limits, map the value of the test statistic *F* (compared to the critical value from the *F*-distribution at various levels of significance) into “causality intensity” between the nodes *i* and *j* as follows:

If the *F* statistic is greater than the critical value at the 0.01 significance level, then 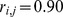;
If the *F* statistic is greater than the critical value at the 0.05 significance level, then 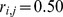;
If the *F* statistic is greater than the critical value at the 0.10 significance level, then 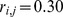;
If the *F* statistic is lower than the critical value at the 0.10 significance level, then 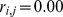.

The fact that the lack of causality translates into a 0 value corresponds to the value from the correlation matrix (zero correlation, resulting from a value lower than 0.3).

## Results

### Data Used

We focus on the most significant economies using two different sets, the first one being the set of economies in the G7 group, consisting of the USA, France, Germany, United Kingdom, Italy, Japan and Canada. The second group consists in economies from OECD. Several economies are excluded since the sample data is available starting only from 1990s and there are newer entrants into OECD. The list of the OECD economies included in the sample is presented in Table 1, which also lists the abbreviation used for each economy, including the countries from the G7 group.

**Table 1:** Countries from OECD included in the sample and their abbreviations.

Countries	Abbreviation
Australia	AUS
Austria	AUT
Belgium	BEL
Canada	CAN
Chile	CHL
Denmark	DNK
Finland	FIN
France	FRA
Germany	GER
Greece	GRC
Hungary	HUN
Iceland	ISL
Ireland	IRN
Israel	ISR
Italy	ITA
Japan	JAP
Korea	KOR
Luxemburg	LUX
Mexico	MEX
Netherlands	NLD
New Zealand	NZL
Norway	NOR
Poland	POL
Portugal	PRT
Spain	ESP
Sweden	SWE
Switzerland	CHE
Turkey	TUR
United Kingdom	GBR
United States	USA

**Figure 1:**
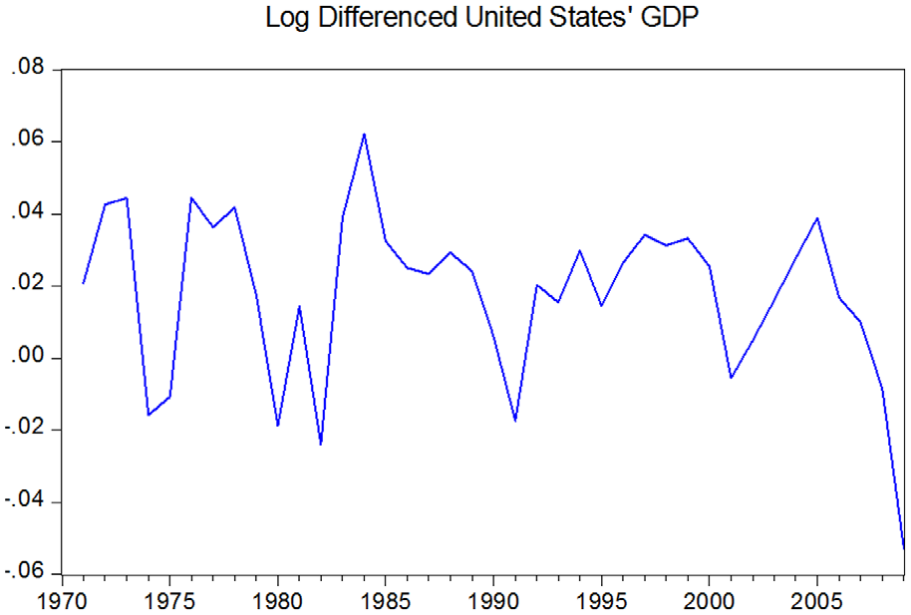
Log-differenced US GDP.

The data were taken from the freely available international macroeconomic data Penn World Table (http://pwt.econ.upenn.edu/php_site/pwt_index.php) at University of Pennsylvania and consists in per capita GDP converted at power purchasing parity in 2005 constant prices. The sample was drawn between 1970 and 2009 and it consists of yearly GDP data. The figures with the rates of growth for each country can be found in the Materials S1. See also Figure 1 for the data on US economy.

The Granger causality approach is conditional on the fact that the two series are stationary. If this condition is not fulfilled, the results have no meaning. We tested for stationarity for each series using the Augmented Dickey Fuller test. The results are presented in Table 2

**Table 2:** Tests for stationarity for differenced and HP filtered series.

Countries	Differenced series	HP filtered series
Australia	−5.92*	−3.77*
Austria	−5.69*	−4.28*
Belgium	−5.25*	−3.50**
Canada	−3.18**	−3.72**
Chile	−4.62*	−3.81*
Denmark	−4.26*	−4.10*
Finland	−3.63*	−4.58*
France	−3.51**	−3.60**
Germany	−3.93*	−4.93*
Greece	−4.81*	−4.42*
Hungary	−2.38	−3.03**
Iceland	−4.86*	−3.70*
Ireland	−3.90*	−3.82*
Israel	−4.66*	−4.78*
Italy	−3.70*	−4.11*
Japan	−2.89***	−4.59*
Korea	−5.32*	−3.69*
Luxemburg	−3.71*	−4.22*
Mexico	−4.06*	−4.28*
Netherlands	−3.28**	−3.50**
New Zealand	−4.27*	−4.00*
Norway	−3.03**	−4.16*
Poland	−3.30**	−4.41*
Portugal	−4.29*	−5.19*
Spain	−1.98	3.98*
Sweden	−3.28**	−4.25*
Switzerland	−4.83*	5.04*
Turkey	−5.76*	−4.89*
United Kingdom	−3.05**	−3.84*
United States	−3.49**	−4.71*

With the exception of Hungary and Spain, the null of unit root is rejected for all cases. We decided to eliminate both Hungary and Spain from the construction of networks based on rates of growth (stationarity is not necessary for correlation, however we would like to make possible the comparison between the correlation networks and Granger causality networks). We also notice that when the series are filtered with the HP filter, the null of unit root is rejected for all cases, including Hungary and Spain.

### Correlation Based Matrices

We first consider the construction of complex networks based on simple correlations. The correlations (and cross-correlations) are widely used in the business cycles analysis and they are part of the standard way to characterize business cycles properties apart from the standard deviation, see [14]. At the same time it is a known fact that the resulting business cycles facts are sensitive to the detrending procedure.

We construct two correlations matrices for each of the data set using both the growth rates and the Hodrick Prescott filtered series. We set a threshold value for statistical significance at 0.3. That is, if the correlation between the nodes *i* and *j* is lower or equal to 0.30, it is set at zero.

**Figure 2:**
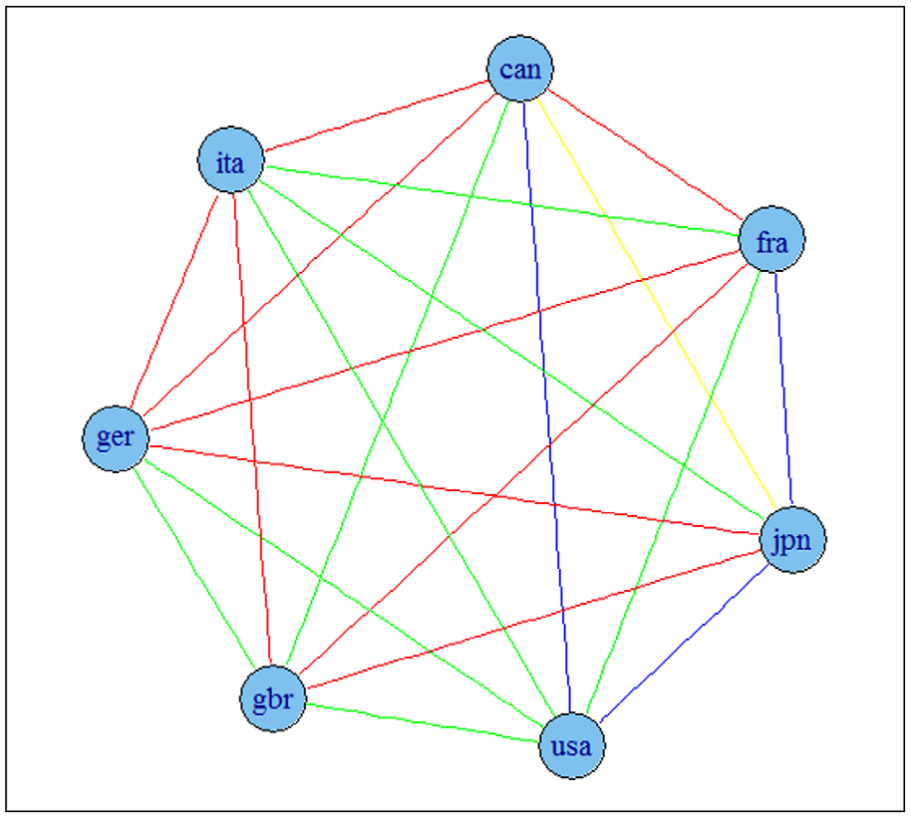
Correlation based network for G7 economies using growth rates of series. Note: colours corresponds to the weights according to the strength of the correlation, namely black for a value higher than 0.9, blue for a value between 0.75 and 0.9, green for a value between 0.6 and 0.75, red for a value between 0.45 and 0.6 and yellow for a value between 0.3 and 0.45.

**Figure 3:**
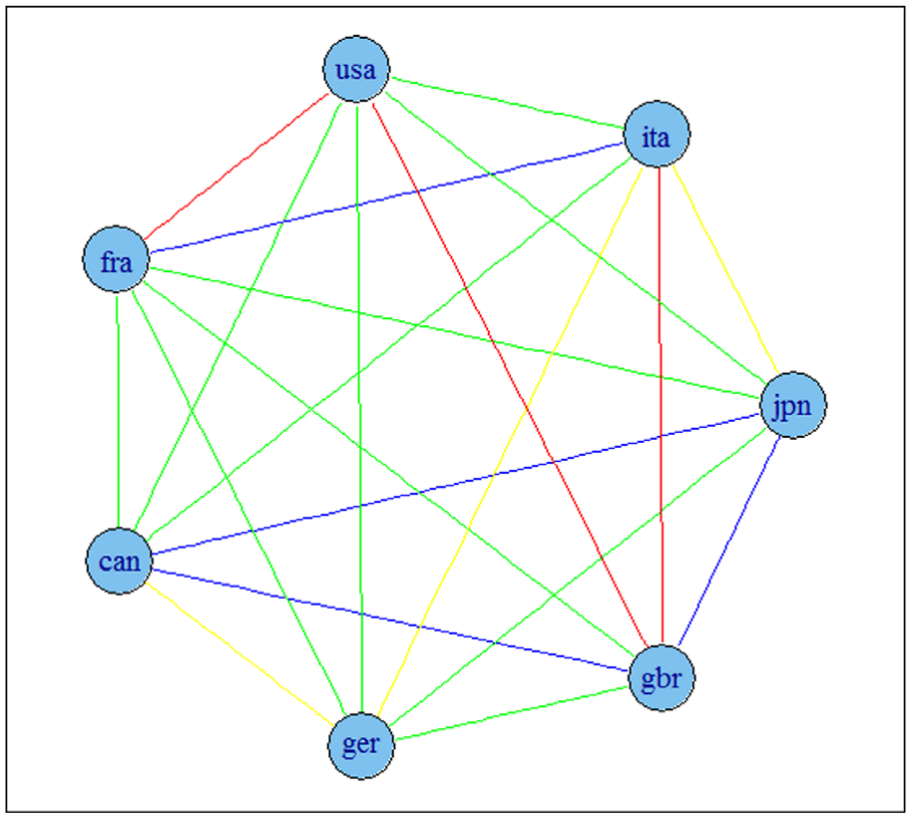
Correlation based network for G7 economies using HP filtered series. Note: colours corresponds to the weights according to the strength of the correlation, namely black for a value higher than 0.9, blue for a value between 0.75 and 0.9, green for a value between 0.6 and 0.75, red for a value between 0.45 and 0.6 and yellow for a value between 0.3 and 0.45.

**Figure 4:**
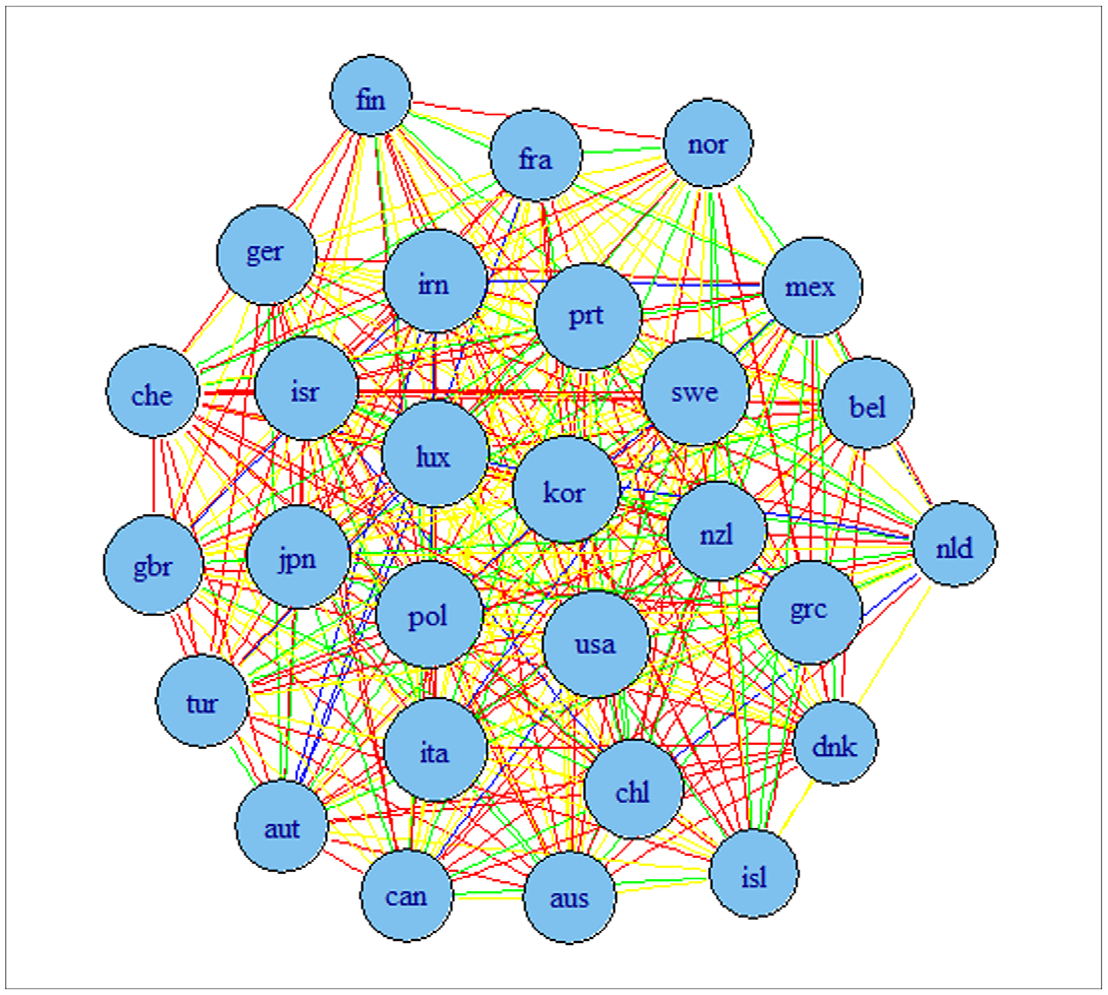
Correlation based network for OECD economies using growth rates of series. Note: colours corresponds to the weights according to the strength of the correlation, namely black for a value higher than 0.9, blue for a value between 0.75 and 0.9, green for a value between 0.6 and 0.75, red for a value between 0.45 and 0.6 and yellow for a value between 0.3 and 0.45.

**Figure 5:**
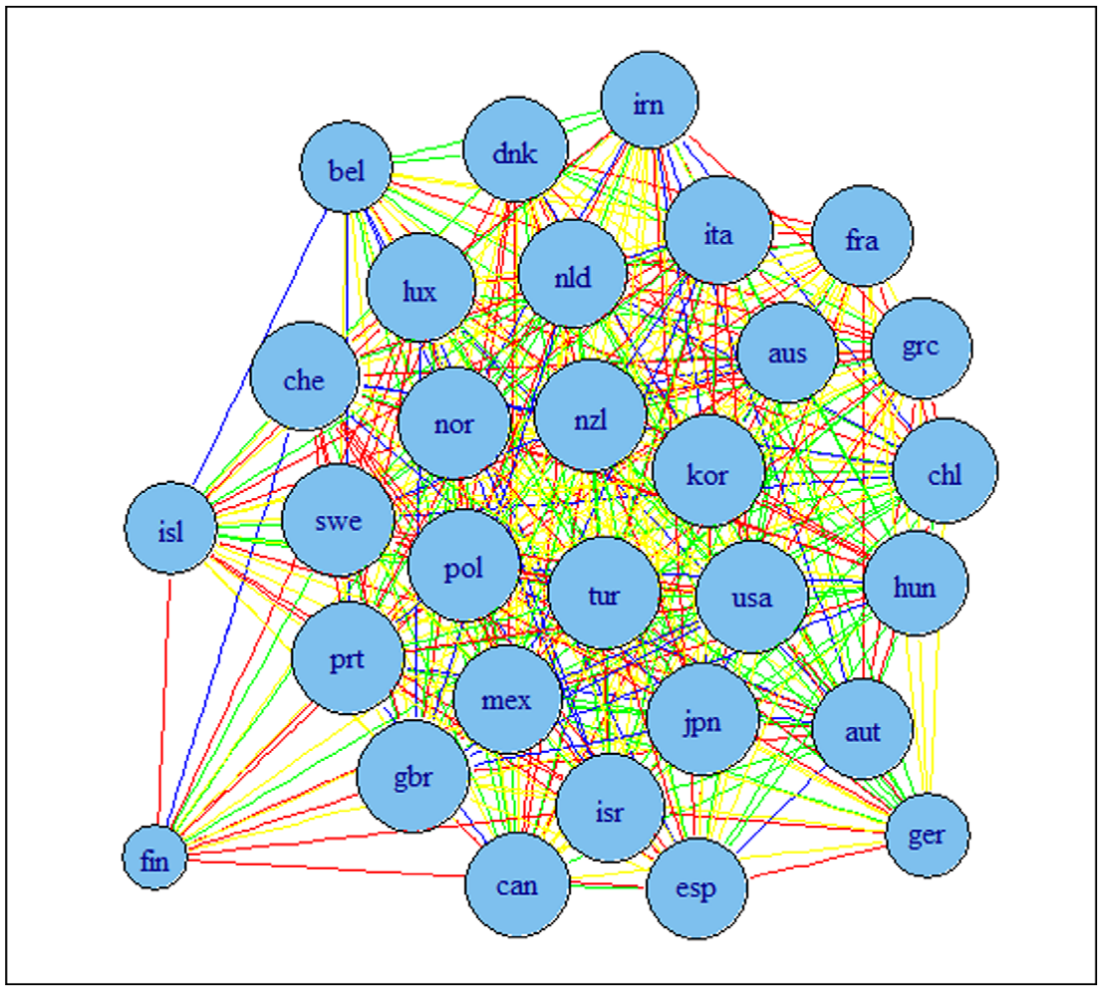
Correlation based network for OECD economies using HP filtered series. Note: colours corresponds to the weights according to the strength of the correlation, namely black for a value higher than 0.9, blue for a value between 0.75 and 0.9, green for a value between 0.6 and 0.75, red for a value between 0.45 and 0.6 and yellow for a value between 0.3 and 0.45.

The results are presented in Figures 2 and 3 for G7 data and Figures 4 and 5 for OECD data. The results indicate that the detrending methods influences the derived complex network. There are a number of changes, between the graphs for OECD economies when using growth rates or HP filtered series.

### Granger Causality Based Complex Networks

We applied a Granger causality approach to derive complex networks of national economies. This is clearly a stronger concept than the correlation one. This approach addresses the shortcoming of the correlation based method to construct complex networks. The most important change is the fact that, based on Granger causality, one can obtain directed networks.

In implementing this approach we consider testing for the optimal lag length. In doing so, we consider up to eight lags, since we are interested in detecting comovement at the business cycle level and business cycles happen with a frequency between 4 and 8 years. In selecting the maximal number of lags we also have to take into consideration the limited sample of only 40 observations. The optimal lag length is chosen using the Bayesian Information Criterion. The selected optimal lag lengths for each case are available in the Materials 2. The F statistics of the Granger causality test are also available as Materials 3.

**Figure 6:**
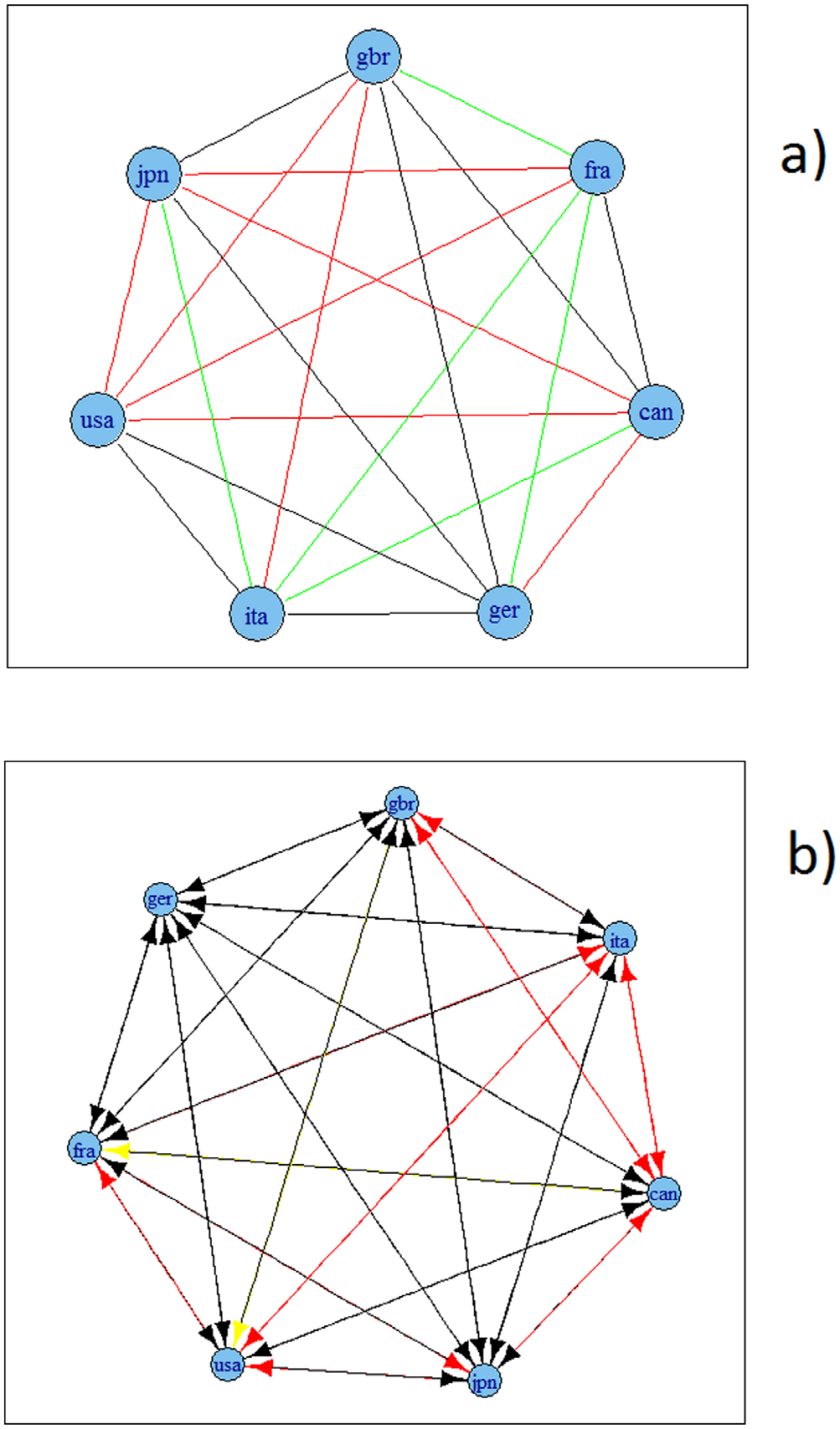
Granger causality based network for G7 economies using growth rates of time series. A: undirected. B: directed. Note: colours corresponds to the weights according to the strength of the correlation, namely black for a value higher than 0.9, blue for a value between 0.75 and 0.9, green for a value between 0.6 and 0.75, red for a value between 0.45 and 0.6 and yellow for a value between 0.3 and 0.45.

**Figure 7:**
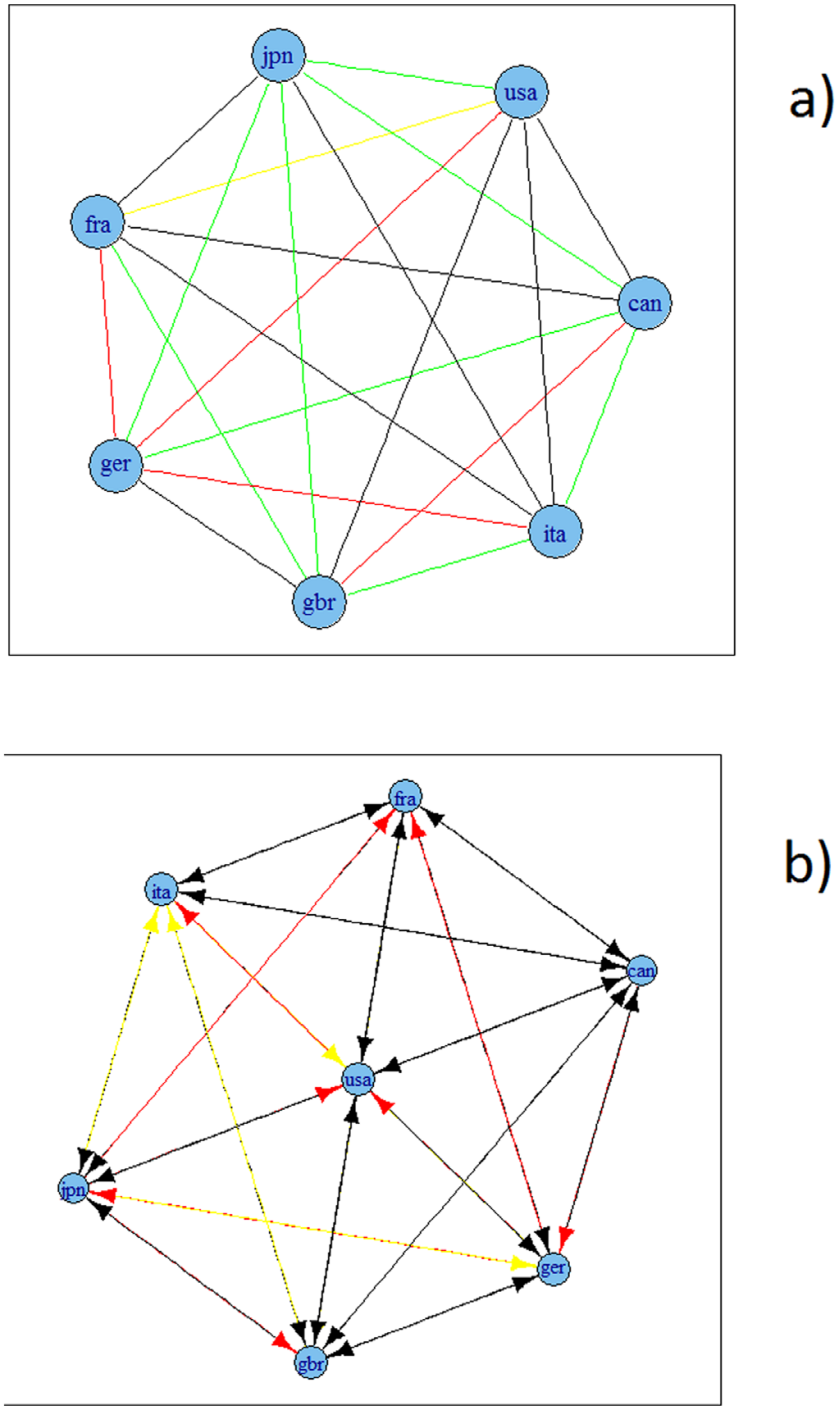
Granger causality based network for G7 economies using HP filtered series. A: undirected. B: directed. Note: colours corresponds to the weights according to the strength of the correlation, namely black for a value higher than 0.9, blue for a value between 0.75 and 0.9, green for a value between 0.6 and 0.75, red for a value between 0.45 and 0.6 and yellow for a value between 0.3 and 0.45.

**Figure 8:**
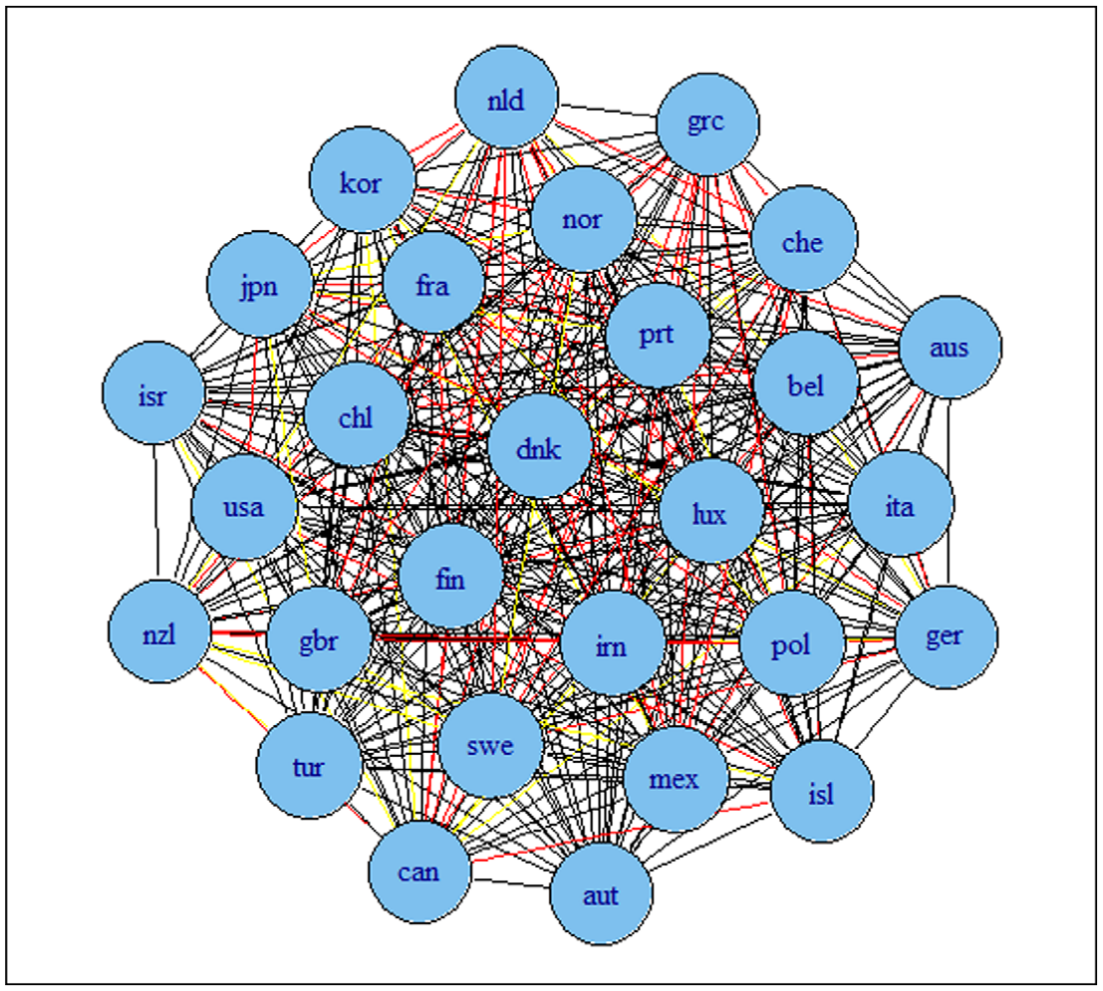
Granger causality based network for OECD economies using growth rates of time series. Note: colours corresponds to the weights according to the strength of the correlation, namely black for a value higher than 0.9, blue for a value between 0.75 and 0.9, green for a value between 0.6 and 0.75, red for a value between 0.45 and 0.6 and yellow for a value between 0.3 and 0.45.

**Figure 9:**
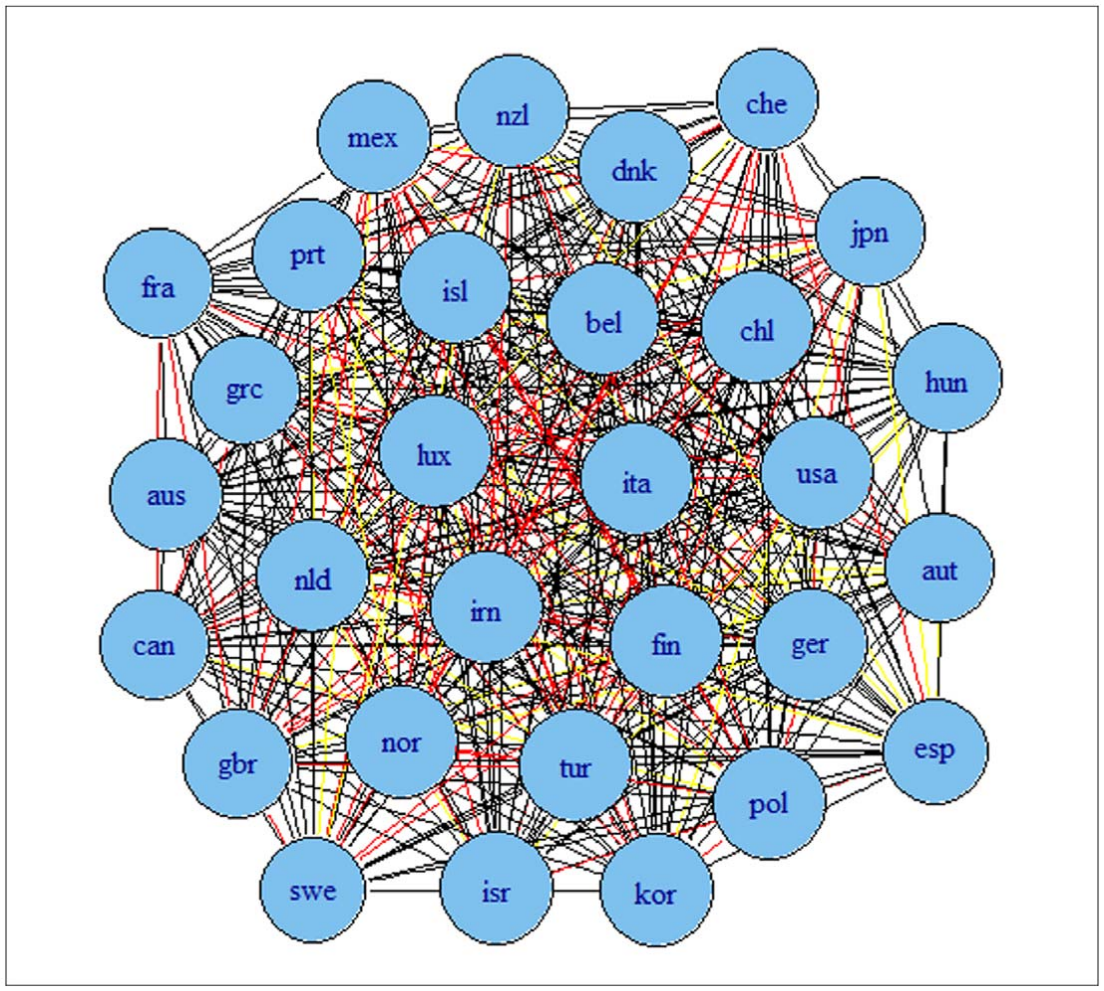
Granger causality based network for OECD economies using HP filtered series. Note: colours corresponds to the weights according to the strength of the correlation, namely black for a value higher than 0.9, blue for a value between 0.75 and 0.9, green for a value between 0.6 and 0.75, red for a value between 0.45 and 0.6 and yellow for a value between 0.3 and 0.45.

Figures 6, 7, 8 and 9 present the resulting graphs, Figures 6a and 7a for G7 economies and Figures 8 and 9 for OECD. We have also considered undirected graphs for G7 economies, Figures 6b and 7b, while for the OECD economies, Figures 8 and 9, we have considered only undirected graphs since it was very difficult to visualize all relationships with a directed graph.

### Relative Influence of Different Nodes

In order to gain some insight into the features of the resulting network we use the approach proposed in [3] to derive measures of the influence of the different nodes (countries) in the Granger causality-based network for G7 and OECD economies, since based on this approach we could obtain directed networks. We focus on the case of HP filtered series since in this case a maximal number of economies is included in the sample.

Given that we have obtained weighted (directed) networks, the following formula is used to determine the relative influence measure of each node:
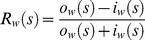
(5)Where *o_w_(s)* is the weighted number of out-degrees link (links going from a certain country *s* to other countries), while *i_w_(s)* is the weighted number of in-degree links (links from other countries to the same country *s*).

The results of applying this method are presented in Table 3, for the G7 sample, and in Table 4 for the OECD economies. Since the size of the economy surely determines the influence of an economy on the business cycles of another economy, we have also weighted the resulting values with the share of each country GDP's in the US GDP (in per capita terms, as mentioned above).

**Table 3:** Weighted Relative influence of countries in G7 sample in Grangers causality based network (HP filtered series).

Country	R_w_	Relative GDP to US (per capita)	Relative Influence
USA	0.524	100.00	52.38
France	0.157	17.93	2.80
Canada	0.171	9.30	1.59
Italy	−0.129	15.99	−2.06
UK	−0.195	17.28	−3.36
Germany	−0.135	25.80	−3.48
Japan	−0.256	32.69	−8.36

**Table 4:** Weighted Relative influence of countries in OECD in Grangers causality based network (HP filtered series).

Country	R_w_	Relative GDP to US (per capita)	Relative Influence
*USA*	0.237	100.000	23.737
*Ireland*	0.195	15.990	3.110
*Spain*	0.152	17.931	2.734
*Australia*	0.145	6.629	0.959
*Poland*	0.172	3.205	0.550
*Portugal*	0.145	3.064	0.444
*Chile*	0.165	1.728	0.285
*Switzerland*	0.181	1.339	0.242
*Belgium*	0.073	3.275	0.238
*France*	0.101	2.358	0.238
*Norway*	0.103	1.659	0.171
*Denmark*	0.091	1.428	0.130
*Greece*	0.072	1.023	0.074
*Netherlands*	0.009	4.752	0.042
*Canada*	0.003	9.306	0.028
*Italy*	0.073	0.260	0.019
*UK*	−0.005	1.623	−0.008
*Germany*	−0.121	0.092	−0.011
*Austria*	−0.069	2.642	−0.182
*Luxembourg*	−0.238	1.007	−0.239
*Iceland*	−0.020	32.696	−0.652
*Finland*	−0.026	25.809	−0.679
*Israel*	−0.125	5.706	−0.711
*Hungary*	−0.694	1.144	−0.794
*New Zealand*	−0.085	9.948	−0.843
*South Korea*	−0.208	5.520	−1.146
*Sweden*	−0.306	4.813	−1.471
*Turkey*	−0.085	17.288	−1.476
*Japan*	−0.160	9.304	−1.490
*Mexico*	−0.864	1.747	−1.509

## Discussion

The emerging field of complex networks has expanded quickly, see [19], showing promising results in many fields including economics, although most of the research focused on financial markets.

In this paper we researched the less studied topic of international business cycles, by characterizing the comovement of national economies using complex network. Besides the known correlation based approach we also introduced the use of Granger causality based networks.

The first important finding is that the derived networks are sensitive to the detrending method used, an issue of particularly importance in macroeconomics, although it might be significant for finance too as constructing correlation-based networks has become a standard tool. The results might be further compared to different methods to construct complex networks.

Another salient issue is how to interpret the resulting networks. In financial markets applications so far, we have seen that there is the tendency of stock markets to group along the economic sectors they are part of. There are some reasons we should not expect a similar result here.

1. The stock markets from advanced economies (with most of the applications done for traded stock on the New York Stock Exchange) generally have certain comovement patterns while the national economies have a much more heterogeneous behavior over time;
2. For the case of comovement between economies, we might expect some smaller economies to co-move in a stronger manner with developed economies;
3. There is no corresponding concept for a sector at the international level although some studies have constructed networks on the global economy using data on trade (See the introduction section.).

A third issue is related to the way networks are constructed. In correlation networks, we would expect the distance between countries to express the degree of comovement, while for Granger causality based networks, the relationship between the nodes expresses the ability of a certain country's GDP to forecast the future changes in the other country's GDP.

In the end, the fact that a certain country, even a small open economy one, is more “central” to the network than we would expect, does not imply that this country has a strong influence on or is a better predictor of world business cycles. Rather, it strongly commoves with the world business cycle for the correlations networks. We should actually expect some small open economies to be more “central”, since they are more vulnerable to changing global economic conditions.

In order to better understand the results, we ordered the nodes (countries) in the Granger causality based networks for the HP filtered series according to their influence scores. (See Tables 3 and 4). In both samples, the most influential node is that of United States, clearly an expected result.

The OECD graph shows a number of small open economies, more developed like Australia, Ireland or Spain or less developed like Poland and Portugal, having also a strong influence on the network. This would rather suggest that these small open economies are good predictors for the changes in international GDPs at the business cycle level. One possible explanation for this somewhat unexpected result is that the smaller and well-connected economies are more sensitive to international shocks and more prone to over respond to changing world business conditions than large economies, like the United States, which can buffer shocks better. When taking into account the relative GDP to US of each country, the results do not change too much. Somewhat of a surprise is the way some members of the called PIGS club (Portugal, Ireland, Greece, Spain) are among the more “influential” nodes, a fact that underlines their strong linkage with the world business cycle (as underlined during the last financial crisis). This does not imply that these countries “lead” the business cycles, although a default of one of the PIGS countries would trigger negative effects on even more developed economies.

Our results are similar to those in [12] who observed the existence of small positive correlations among his sample of 20 industrialized economies. These results also point to the difficulty of analyzing the business cycles with complex networks. Although the complex networks have a great potential in analyzing the international business cycles, there are certain limitations that should be overcome in the future. Research should take into consideration a wider range of factors that might help picturing better the international business cycles (such as financial variables).

Besides the obvious application of the results in this paper to the analysis of business cycles, further applications may include the construction of risk indices with the help of network theory which would take into account the macroeconomic factors or the analysis of the interaction between the macroeconomic and the financial networks. In the end the results here can be further developed to construct model of crisis transmission within a network framework as previous work by [8] has suggested.

## Supporting Information

Materials S1 **This file contains the figures with the growth rates of the GDP of each country in the sample.**
(XLS)

Materials S2 **This file contains information regarding the optimal lag lengths as detected by the Granger causality tests between country i (on rows) and country j (on columns).** Diagonals’ values are missing since we do not run causality tests between a time series and itself. There are two sheets, one for the data in growth specification (log differenced) and one for HP filtered data, namely Growth data sheet and HP filtered data sheet. The abbreviations correspond to the names which can be found in Table 1.
(XLS)

Materials S3 **This file contains information regarding the F statistics as produced by the Granger causality tests between country i (on rows) and country j (on columns).** Diagonals’ values are missing since we do not run causality tests between a time series and itself. There are two sheets, one for the data in growth specification (log differenced) and one for HP filtered data, namely Growth data sheet and HP filtered data sheet. The abbreviations correspond to the names which can be found in Table 1.
(XLS)

## References

[1] Mantegna RN (1999) Hierarchical structure in financial markets. Eur. Phys. J. B 11, 193–197.

[2] Tumminello M, Aste T, Di Matteo T, Mantegna RN (2005) A Tool for Filtering Information in Complex Systems. Proceedings of the National Academy of Sciences 102 (30), 10421–10426. 10.1073/pnas.0500298102 PMC1180754 16027373

[3] Kenett DY, Tumminello M, Madi A, Gur-Gershgoren G, Mantegna RN, et al.. (2010) Dominating Clasp of the Financial Sector Revealed by Partial Correlation Analysis of the Stock Market. Plos One 5, 15032. 10.1371/journal.pone.0015032 PMC3004792 21188140

[4] Kenett DY, Shapira Y, Madi A, Bransburg-Zabary S, Gur-Gershgoren G, et al.. (2011) Index Cohesive Force Analysis Reveals That the US Market Became Prone to Systemic Collapses Since 2002. Plos One 6(4), e19378. 10.1371/journal.pone.0019378 PMC3083438 21556323

[5] Kenett DY, Preis T, Gur-Gershgoren G, Ben-Jacob E (2012) Dependency network and node influence: application to the study of financial markets. International Journal of Bifurcation and Chaos 22, 1250181.

[6] Kenett D, Raddant M, Lux T, Ben-Jacob E (2012) Evolvement of uniformity and volatility in the stressed global financial village. Plos One (7), e31144. 10.1371/journal.pone.0031144 PMC3275621 22347444

[7] Kali R, Reyes J (2010) Financial contagion on the international trade network. Economic Inquiry 48, 1072–1101.

[8] Lee K-M, Yang J-S, Kim G, Lee J, Goh K-I, et al.. (2011) Impact of the Topology of Global Macroeconomic Network on the Spreading of Economic Crises. Plos One 6(3), e18443. 10.1371/journal.pone.0018443 PMC3069097 21483794

[9] Lucas RE (1977) Understanding business cycles. Carnegie-Rochester Conference Series on Public Policy 5, 7–29.

[10] Backus D, Kehoe P (1992) International Evidence on the Historical Properties of Business Cycles. American Economic Review 82, 864–888.

[11] Backus D, Kehoe P, Kydland F (1995) International Business Cycles: Theory and Evidence, in Thomas F. Cooley, ed., Frontiers of Business Cycle Research (Princeton, NJ, Princeton University Press), 331–356.

[12] Ambler S, Cardia E, Zimmermann C (2004) International business cycles: What are the facts? Journal of Monetary Economics, 51, 257–276.

[13] Kenett DY, Preis T, Gur-Gerschgoren G, Ben-Jacob E (2012) Quantifying meta-correlations in financial markets. EPL 99, 38001.

[14] Hodrick RJ, Prescott EC (1997) Postwar U.S. Business Cycles: An Empirical Investigation. Journal of Money, Credit, and Banking 29, 1–16.

[15] Christiano L, Fitzgerald TJ (2003) The Band Pass Filter. International Economic Review 44, 435–465.

[16] Ramsey J, Lampart C (1998) Decomposition of economic relationships by time scale using wavelets. Macroeconomic dynamics 2, 49–71.

[17] Canova F (1998) Detrending and business cycle facts. Journal of Monetary Economics 41, 475–512.

[18] Granger CWJ (1969) Investigating causal relations by econometric models and cross-spectral methods. Econometrica 37, 424–438.

[19] Albert R, Barabasi AL (2002) Statistical mechanics of complex networks. Review of Modern Physics 74, 47–97.

